# Populations of triple negative and hormone receptor positive HER2 negative breast tumors share immune gene profiles

**DOI:** 10.21203/rs.3.rs-4542494/v1

**Published:** 2024-08-02

**Authors:** Sasha Stanton, Frank Schmitz, Wilbert Copeland, Justine DellAringa, Kathryn Newhall, Mary Disis

**Affiliations:** Earle A. Chiles Research Institute; Fred Hutchinson Cancer Research Institute; Celgene Corporation; Celegene Corporation; Celgene Corporation; University of Washington

**Keywords:** breast cancer, triple negative breast cancer, hormone receptor positive HER2 negative breast cancer, immune infiltrate

## Abstract

In breast cancer, triple negative (TN) breast cancer has most responses to immune checkpoint inhibitor (ICI) therapy. Lymphocyte infiltrate does not impact prognosis in Hormone receptor positive HER2 negative (HR + HER2-) breast tumors and few HR + HER2− tumors respond to ICI. We contrasted immune-associated gene expression between 119 TN and 475 HR + HER2− breast tumors from The Cancer Genome Atlas (TCGA) and confirmed our findings in 299 TN and 1369 HR + HER2− breast tumors in the METABRIC database. TN and HR+ HER2− tumors grouped into immune-high or -low tumors, both subtypes were represented in the immune-high group. The largest difference between the immune-high TN and HR + HER2− tumors was TN tumors had more abundant T_h1_ and T_h2_ CD4^+^ T cells while HR + HER2− tumors had more abundant fibroblasts (log_2_FC > 0.3; *p* < 10×10^−10^). This suggests an immune-high signature is not dictated by breast cancer subtype, but fibroblast subsets associated with worse outcome were higher in the immune-high HR + HER2− tumors.

## Background

The histological subtyping of breast cancer, based on hormone receptors (HR) (estrogen receptor, ER, progesterone receptor, PR) and human epidermal growth factor receptor ERBB2 (HER2) is essential to clinical management, determining prognosis, and treatment of breast cancer.[1–4] The histologic subtypes have differences in the type and amount of immune infiltrate but a spectrum of immune infiltrate exist in all breast cancer subtypes.[5] The benefit of immune checkpoint inhibitors (ICI) have been seen in metastatic cancers including renal cell, melanoma, and non-small-cell lung cancer.[5–7] The predictive biomarkers associated with response to ICI include PD-L1 expression, IFNy expression, and increased somatic tumor mutations.[6, 7] Also, a determinant for response to ICI therapy is the presence of an adaptive immune response within a tumor prior to treatment.[8–11] Breast cancer does not have the same level of adaptive immune infiltrate seen in melanoma and non-small cell lung cancer although the level of immune infiltrate in breast cancer is associated with clinical prognosis.[12] Breast tumors with greater than 50% tumor infiltrating lymphocytes (TIL) show improved pathologic complete response to neoadjuvant chemotherapy (*p* ≤ 0.01)[11] and increased intratumoral CD8 + T cells predict improved overall survival (OS) (*p* ≤ 0.01).[13] In the adjuvant setting, each 10% increase of TIL predicted a 15% improved disease free survival (DFS) (*P-value* = 0.025) and 17% improved OS (*P-value* = 0.023) in TN but not HR+ HER2− breast cancer.[14, 15]

In clinical studies, response to ICIs have best been seen in metastatic TN breast cancer which had a 19% response rate to pembrolizumab as a monotherapy.[16] In contrast, metastatic HR+ HER2 breast cancer either with pembrolizumab as a single agent (KEYN0TE-028) or in combination with a CDK4/6 inhibitor had an overall response rate of ~ 12%.[17, 18] In neoadjuvant therapy, pembrolizumab has improved pathologic complete response (pCR) in both HR + HER2- and TN. However, while the addition of pembrolizumab improved pCR in HR+ HER2− tumors from 15.6–24.3%, the addition of pembrolizumab improves pCR in TN from 51.2–64.8%.[19, 20] The same magnitude of benefit of ICI therapy seen in TN breast cancer was not seen in HR + HER2− breast cancer, leading to the classification of HR + HER2− breast cancer as “cold” tumors that do not respond to immune therapy.

Targeted exome sequencing technology has proved useful for elucidating the complex relationships between cancer and the immune system through RNA expression. For example, HTG Oncology’s immune-associated gene set was used to identify ICI therapy responders across a wide variety of cancers including urothelial, colon, and lung [21, 22] and has been used to identify neoadjuvant ICI response in TN breast cancer in the GeparNeuvo trial.[23] The HTG oncology panel has also been used in breast cancer clinical trials to determine changes in expression that predict treatment effect with palbociclib in PALOMA2 and PALOMA3.[24] Therefore HTG gene sets have been used to investigate both immunologic and oncologic gene expression patterns in cancer that predict clinical responses.

We questioned whether we could identify distinct immune gene expression patterns in HR+ HER2- and TN breast cancers that could account for the differences in immune infiltrate between these subtypes. Analyzing gene expression data from 594 breast cancer samples using the HTG Oncology and Immuno-oncology panels, 119 TN and 475 HR + HER2− from The Cancer Genome Atlas (TCGA), we found two distinct subgroups of immune-high and immune-low tumors in both subtypes. This was confirmed in 1668 HER2− tumors in the METABRIC dataset, 299 TN and 1369 HR+ HER2− breast tumors. After estimating relative cellular phenotype abundances from bulk RNA sequencing, we found cancer-associated fibroblasts more abundant in HR + HER2− tumors, particularly higher in immune-high HR + HER2− tumors than immune-high TN tumors, while helper T cell populations were more abundant in immune-high TN tumors. These differences between immune high TN and HR + HER2− tumors may contribute to the differences in response to ICI but further work is needed to determine if CAF may provide targets to improve HR + HER2− response to ICI.

## Materials and Methods

### Collection of datasets from The Cancer Genome Atlas (TCGA)

Normalized, bulk RNA sequencing expression data and clinical annotations were downloaded from the UCSC Xena TCGA portal for 119 TN and 475 HR + HER2− (412 ER+ PR+, 63 ER + PR-, 0 ER-PR+) breast cancer patients. Therefore the TCGA analysis set included 20% TN and 80% HR + HER2− tumors. HR + HER2- and TN subtypes were defined using the clinical guidelines from the American Society of Clinical Oncology and are publicly available from previous studies of breast tumors in TCGA.[25] Mutation data (MAF) files were downloaded from the GDC Data Portal and mutational burden was analyzed using maftools.[26] The METABRIC gene expression and annotation was downloaded from the cBioPortal for Cancer Genomics and included 1369 HR + HER2- and 299 TN tumors. Annotations for immune subtypes (C1-C6) were collected from Thorsson *et al*.[27] Annotations for the fibroblast subtypes were collected from Bartoschek *et al*[28]

### Oncology- and immune-associated gene sets

RNA expression was reported for 20,530 genes. We divided genes into two categories: oncology- and immune-associated (Table S1A–B). 2,500 were captured in the HTG Edge Seq Oncology Biomarker and 543 genes were captured in the HTG Immune assays (HTG Molecular Diagnostics, Inc., Tucson, AZ).

### K-means clustering and silhouette analysis

To determine the optimal number of clusters for k-means, silhouette analysis (from the R ‘cluster’ and ‘factoextra’ packages) was performed on the onco- and immune-associated genes independently. K-means clustering was performed (K = 2; Hartigan and Wong algorithm, ‘kmeans’ from the R ‘stats’ package) on RNA expression values for onco- and immune-associated genes groups separately. Using Silhouette analysis identified k = 2 as optimal number of clusters using the HTG immune genes and oncogene sets (**Fig. S3A-D**). Hierarchical clustering of the most variable genes in the immune-associated gene set reveals two immune-associated gene expression patterns.

### Gene set variation analysis (GSVA) and cell type frequency estimation

GSVA scores were calculated to measure gene set variation relative to the variation observed for all genes. To calculate sample-wise enrichment of a gene set, we used ‘GSVA’ in R.[29] Cell type enrichment was estimated with the ‘xCell’ package in R.[30] We restricted our analysis to immune relevant cell types (Table 1). To confirm xCell enrichment scores, we calculated GSVA scores for independent gene sets for CD8^+^ T cells (CD8A, CD8B, CD3D) or T_reg_ (IL2RA, CCR4, CTLA4, CD28, ICOS, FOXP3, CD5).

### Differential gene expression and cell enrichment

To identify differentially expressed genes or differentially abundant cell types between two groups we performed similar tests. We used *limma* in R[31] to calculate the log_2_fold change (log_2_FC) which describes the differences in gene expression or cell-type frequency between the designated groups. *P*-values were corrected for multiple hypothesis testing using the “BH” (Benjamini Hochberg, controlling the false discovery rate) method.

## Results

### Immune-associated gene expression is not driven by clinical subtype

**Figure 1:**
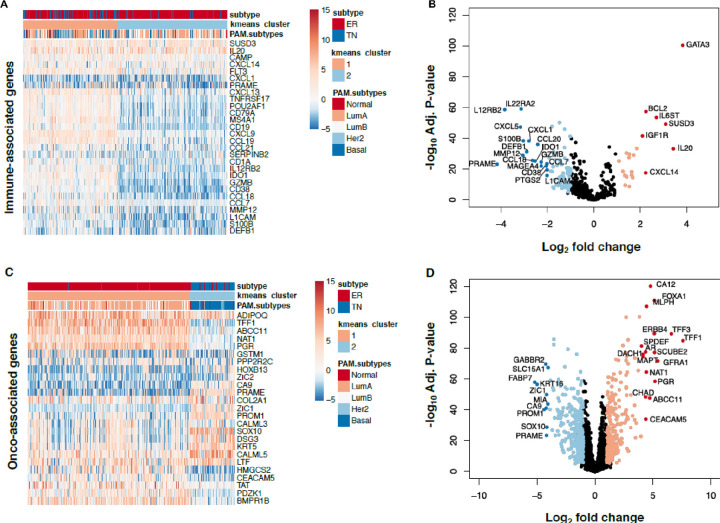
Transcriptomic clustering of immune- and onco-associated genes in HR+HER2- and TN breast tumors. Heatmaps show log_2_ gene expression for 594 tumors (y-axis). The x-axis contains the most variably expressed 25 and 28 genes in immune- and oncology-associated gene subgroups, respectively (**A,C**). Heatmap annotation tracks include K-means (K=2) cluster assignments, generated from either all immune-associated or onco-associated genes. Volcano plots show relationships between log_2_ fold change (FC; x-axis) and Benjamini-Hochberg adjusted *P*-values (y-axis) for differentially expressed genes in HR+HER2- and TN subtypes using immune-associated (**B**) or oncology associated (**D**) genes. Differentially expressed genes more highly expressed in HR+HER2− tumors are red and pink, while genes more highly expressed in TN tumors are blue and light blue. In 1B, the cutoffs are (Adj. *P*-value<0.05; abs(Log_2_FC)>2) for red and blue, and (Adj. *P*-value<0.05; 1 < abs(Log_2_FC) <2) for pink and light blue. In 1D, the cutoffs are (Adj. *P*-value<0.05; abs(Log_2_FC)>4) for red and blue, and (Adj. *P*-value<0.05; 1 < abs(Log_2_FC) <4) for pink and light blue.

To investigate RNA expression profiles of breast cancer samples, we analyzed 119 TN and 475 HR + HER2− breast cancer samples and limited our analysis to the genes included in HTG Oncology’s immune and oncology gene sets. For the immune-associated genes, we began by performing k-means clustering to determine the optimal number of groups to immunologically characterize breast tumors (k = 2; see Material and Methods and **Fig S3A and B**). Cluster 1 had higher median log_2_ expression of 543 immune genes than Cluster 2 (0.29 vs. −0.32), and thus Cluster 1 was referred to as ‘immune-high’ and Cluster 2 as ‘immune-low’ (Fig. 1A). Cluster 1 contains 276 breast tumors, 84 TN tumors and 192 HR + HER2− tumors (30% of the immune-high tumors are TN and 70% of the immune-high tumors are HR + HER2-). Cluster 2 contains 318 breast tumors, 35 TN and 283 HR + HER2. When we evaluated which immune-associated genes were differentially expressed between HR + HER2- and TN breast tumors within the immune-high cluster, GATA3 (logFC = 3.8; adj. *P*-value = 4×10^−101^) was most expressed in HR + HER2− tumors (Fig. 1B). GATA3 has previously been associated with hormone receptor expression and lower tumor grade.[32]

We evaluated the same TN and HR + HER2− tumors using the HTG oncology-associated genes to show these tumors genetically matched the breast cancer subtypes found by immunohistochemistry. We performed k-means clustering of 2,500 oncology-associated genes and found that, as expected, there were two major clusters: Cluster 1 is 97% HR + HER2− tumors (461 tumors) and Cluster 2 is 88% TN tumors (107 tumors) (Fig. 1C). The oncology-associated genes overexpressed more in HR+ HER2− are hormone related including ESR1, ERBB4, and PGR which were more expressed in cluster 1 (log FC > 5, P-value < 0.001 for each) (Fig. 1D).

**Figure 2:**
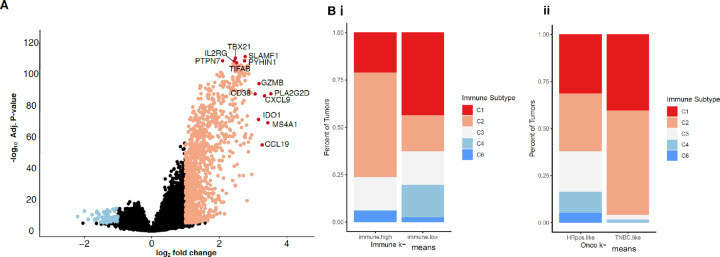
Immune gene and functional differences between immune-high and immune-low breast cancers. The volcano plot shows the log_2_FC (x-axis) and Benjamini-Hochberg adjusted *P*-values (y-axis) for a test of differential gene expression on 20,530 genes in immune-high and immune-low clusters (**A**). Differentially expressed genes more highly expressed in immune-high (cluster 1) tumors are red (Adj. *P*-value<0.05; Log_2_FC>2 or 4.5) and pink (Adj. *P*-value<0.05; Log_2_FC>1) and immune-low (cluster 2) are blue(Adj. *P*-value<0.05; Log_2_ fold change (FC<−2 or 4.5) and light blue (Adj. *P*-value<0.05; Log_2_FC<−1). Bar plots show the relative frequency of immune-subtypes from Thorsson *et al*. (2018). C1–6 are described as follows: wound healing, interferon gamma dominant, inflammatory, lymphocyte depleted, immunologically quiet (not present here) and TGF-β dominant (**B**). **Bi** shows the difference between immune high and immune low groups and **Bii** shows the difference between HR+HER2- and TN groups.

We also performed principal component analysis (PCA) on the oncology and immune-associated gene sets individually. For the oncology-associated genes, the principal component capturing the greatest variance (PC1: 16.4%) between tumor gene expression strongly separates TN and HR + tumors (**Fig. S1A**). This is illustrated by an high area under the curve (AUC) of 0.96 (**Fig. S1B**). However, in a PCA with immune-associated genes, we find the first principal component (PC1: 32%) is less associated with breast cancer subtype than the second principal component (PC2: 8%; AUCs = 0.74 vs. 0.91; **Fig. S1C and D**). Thus, the histologic subtype is not the primary driver of immune-associated gene expression. When evaluating all genes available in TCGA (N = 20,530) the gene expression differences between immune-high and low breast tumors included immune response genes including T cell signaling (*e.g.,* IL2RG) and function (*e.g.,* GZMB) (Fig. 2A).

To provide independent validation of the immune-high and immune-low clusters, we evaluated k-means clusters in the METABRIC dataset. The METABRIC dataset includes 1658 HER2− breast tumors: 299 TN and 1369 HR + HER2−. This dataset similarly showed two immune clusters (see Materials and Methods); an immune-high cluster 1 (240 TN and 384 HR+ HER2− tumors) and an immune-low cluster 2 (59 TN and 985 HR + HER2− tumors). In this data set, 80% of TN tumors and 28% of HR + HER2− tumors were in the immune-high cluster (**Fig. S4A**). Like the TCGA data set, when evaluating expression of the oncology-associated gene set, breast tumors generally clustered with their HR+ HER2- or TN subtype similar to results by immunohistochemistry. There were 97% HR+ HER2- and 12% TN in cluster 1 and 87% TN and 3% HR + HER2− in cluster 2 (**Fig. S4B**).

Using immune clusters previously defined in 33 cancer types in TCGA[27], we evaluated if these clusters distributed both within the oncology and immune-k-means clusters (Fig. 2B). We find the immune-high samples are enriched in C2 as compared to immune-low samples (55% vs 19%; Fig. 2B_i_). Immune-high contains 21% of C1 (wound healing) where immune-low contains 44% of C1. The immune subset C5 was absent in the TCGA dataset, as seen in previous studies.[27] When comparing oncology-gene based clustering, TN tumors are enriched for IFNγ dominant (C2) tumors compared to HR + HER2− (Fig. 2B_ii_). This is illustrated by the difference in the proportion of C2 tumors between HR + HER2− (C2 = 31%) and TN (C2 = 55%) oncology k-means clusters. This is concordant with previous reports describing a stronger T cell presence in TN versus HR + HER2−.[12] However, we find the cluster associated with the best overall survival (C3) is almost exclusively found in HR+ HER2− tumors, with 21% of the HR + HER2− tumors having a C3 immune environment compared to 2.5% of the TN tumors. Forty-six percent of the C3 tumors are immune-high and, as only six of the C3 tumors were TN, the majority of immune-high C3 tumors are HR+ HER2− (N = 43, 91% of all immune-high C3 tumors).[27]

#### Inferred cell abundance differences between TN and HR + HER2− tumors within the immune-high cluster and the immune-low cluster.

**Figure 3:**
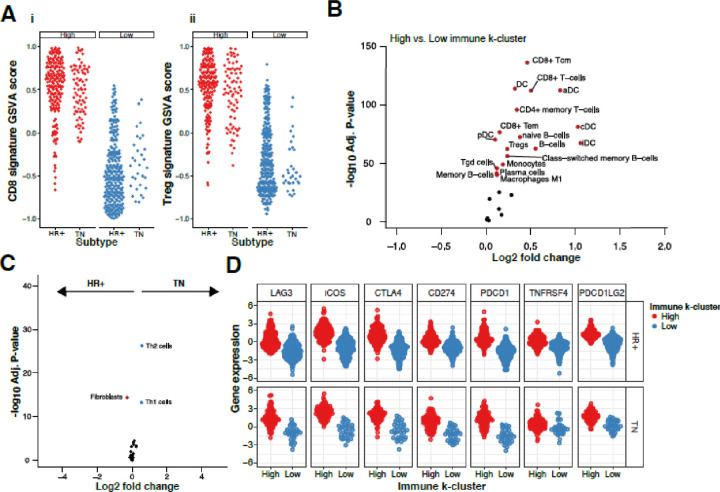
Immune cell differences in immune-high and -low groups. Jitterplots show distributions of CD8+ (**Ai**) and Treg signature (**Aii**) GSVA scores (y-axis). Volcano plot shows the statistical results of a test for differentially abundant cell types in immune-high and -low clusters (**B**). Cell types more highly abundant in immune-high (cluster 1) tumors are red (Adj. *P*-value <10E-40; Log_2_FC>0.1). (**C**) Volcano plot for differential expression of immune-high HR+HER2− (red) and TN (blue) (**D**) Expression of immune regulatory proteins for immune-high (red) and immune-low (blue) tumors that are HR+HER2- or TN (y-axis).

To better understand the underlying differences between TN and HR + HER2− immune-high and -low clusters, we used gene signatures for CD8^+^ (Fig. 3A_i_) and CD4^+^ T_reg_ (Fig. 3A_ii_) T cells as proxy for immune cell type abundance in the tumor. As expected, GSVA scores for CD8^+^ T and CD4^+^ T_reg_ cell signatures in the immune-high cluster were twice that of the immune-low cluster. To estimate immune cell enrichment with cell type signatures, we used xCell[30] and confirmed that xCell scores were behaving appropriately in this dataset including T cells, B cells, dendritic cells, and macrophages that were significantly higher in the immune-high cluster (raw *P*-value <10^− 50^; Fig. 3B).

Next, we sought to characterize the immune differences between TN and HR + HER2− within the patients of the immune-high cluster. Contrasting HR + HER2- and TN in the immune-high cluster, we found TH1 and TH2 CD4 + T cells are more abundant in TN than HR + HER2- and fibroblasts are more abundant in HR + HER2− tumors than TN tumors (log_2_FC > 0.3; *P*-value <10^−10^, Fig. 3C). There was no association between TH1 and TH2 infiltrate in either immune-high (ρ = 0.15; *P*-value = 0.1859) or immune-low (ρ = 0.07 *P*-value = 0.6735) TN breast cancer. However, in HR + HER2− tumors there was a positive association with increased TH1 tumors also having increased TH2 infiltrate in both immune-high (ρ = 29; *P*-value = < 0.0001) and immune-low (ρ = 0.21; *P*-value = 0.0005) (**Fig. S5**). In a similar analysis of the immune-low cluster, we observe more T_h1_ and T_h2_ CD4 + T cells in TN than in HR+ HER2− tumors and more fibroblasts in HR + HER2− tumors than TN tumors (**Fig. S2**). This suggests the immune infiltrate may be impacted by the differences in fibroblasts between HR + HER2- and TN subtype regardless of the tumors being in the immune-high or immune-low groups.

#### Immune checkpoint genes are differentially expressed between TN and HR + HER2− in the immune-high cluster but not in the immune-low cluster

Immune checkpoint genes LAG3, ICOS, CTLA4, PD1 (PDCD1), PDL1 (CD274), PDL2 (PDCD1LG2), and OX40 (TNFRSF4), have been shown to predict responsiveness of tumors to ICI therapy.[33, 34] Testing for differential expression levels of these genes, we observed statistically significant higher expression in the immune-high group than the immune-low group as expected (Fig. 3D, Welch’s 2 sided T-test; adj. *P*-value < 9×10^− 36^). In the immune low group the immune checkpoint target genes LAG3, ICOS, PD-1, and PD-L1 were not statistically different between TN and HR+ HER2− tumors (adjusted *P-value* **>** 0.05) but CTLA4 and OX40 were higher in TNBC than HR + HER2− (adjusted P-value p = 0.016 each). Irrespective of hormone receptor status, immune checkpoint expression was significantly higher in the immune high cluster than in the immune low cluster (adjusted *P-value* **<** 0.001) and in the immune high group there were no significant differences between HR + HER2- and TN tumors.

**Figure 4:**
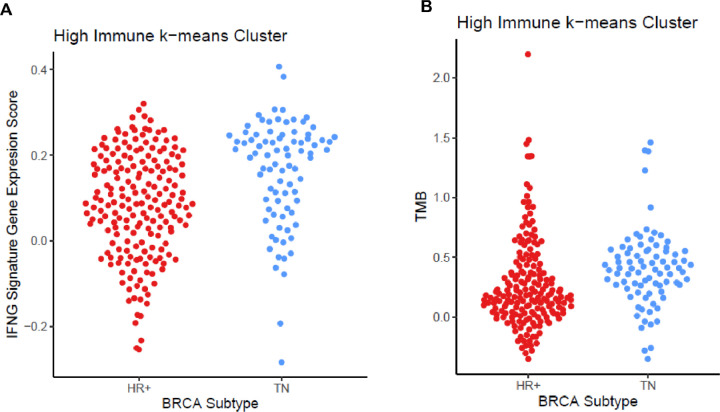
Immunological differences in HR+HER2- and TN breast tumors. Jitterplots show distributions of IFNγ^+^ signature GSVA scores (y-axis; **A**) and TMB (y-axis; **B**) for immune high HR+HER2- and TN tumors (x-axes). TN tumors are blue and HR+HER2− tumors are red.

ICI efficacy is also associated with CD8^+^ T cells tumor infiltration and IFN-γ gene expression patterns in melanoma[35] and lung[6]. We used a gene signature that contains four genes (*i.e*., IFNγ, CD274, LAG3, and CXCL9; IFNγ^+^ signature), previously used to stratify urothelial and non-small-cell lung carcinoma cancer patient response to anti-PD-L1 durvalumab treatment to evaluate the immune-high and immune-low tumors.[6] When evaluating HR + HER2- and TN breast cancer, the mean of the IFNγ^+^ signature score was higher in TN than HR+ HER2− (0.08 vs. −0.047; Welch’s 2 sided T-test *P*-value **=** *5.9×10*^*− 13*^), but the ranges of the scores were overlapping (TN: [−0.32, 0.41]; HR+: [−0.37, 0.32]). Similarly, when evaluating the immune-high group of HR + HER2- and TN tumors, the means of the IFNγ^+^signature scores are different, though less statistically significant (Welch’s 2 sided T-test, *P*-value = *2.2×10*^*− 6*^; Fig. 4A). Notably, when testing the IFNγ^+^ signature score between immune high and low cluster tumors, immune high tumors had significantly higher signatures scores irrespective of the breast cancer subtype (HR+ HER2− Mann-Whitney test, *P*-value = 6.3×10^− 62^, TN Mann-Whitney test, *P*-value = 1.3×10^−18^).

Tumor mutations generate neo-antigens and immune infiltrate can increase with an adaptive immune response to these neo-antigens increasing immune infiltrate. Tumor mutational burden also can determine response to ICI that is tumor-type agnostic.[36] To determine whether tumor mutational load differed between breast cancer subtypes, we calculated each tumor’s tumor mutational burden (TMB). When evaluating the immune high group, the TN tumors contained a significantly higher TMB than the HR + HER2− tumors (Mann-Whitney test, *P*-value = 3.2×10^−15^; Fig. 4B). The difference in the median mutational burden between immune-high and immune-low was also statistically significant (Mann-Whitney test *P*-value = 1.2×10^− 5^) with the immune high tumors having more TMB than the immune low tumors. The relationship between somatic mutations and the IFNγ^+^ signature shows a moderate correlation (Spearman’s ρ = 0.33), indicating that the immune response in breast cancer may in part be driven by somatic mutation derived neoantigens. Furthermore, evaluation of other mutation types (insertion, missense, nonsense) similarly showed significant differences between immune-high HR + HER2- and TN and immune-low HR + HER2- and TN (all p < 0.001, **data not shown**).

#### Fibroblast population associated with worse prognosis in breast cancer are higher in HR + HER2− tumors in both the immune-high and immune-low clusters.

**Figure 5:**
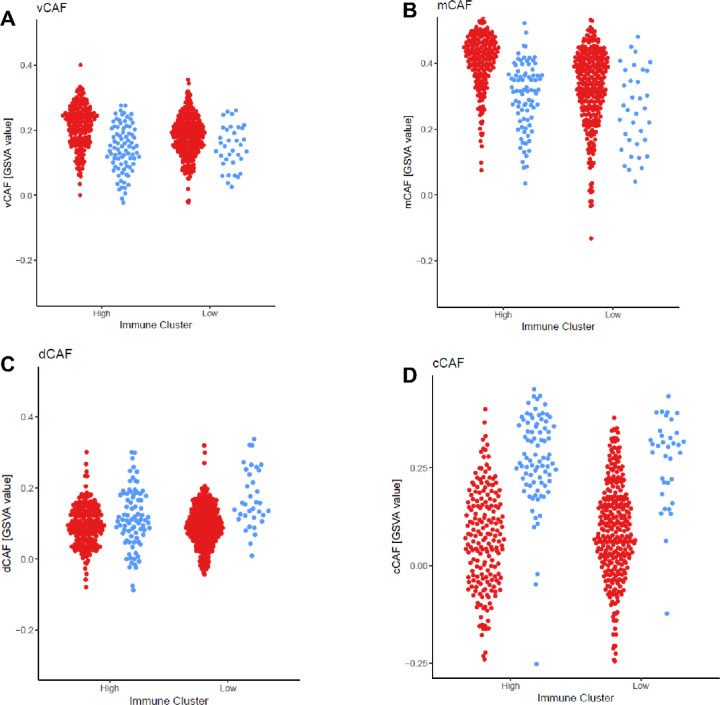
vCAF and mCAF populations are higher in HR+HER2− tumors than in TN breast tumors. GSVA scores for four cancer associated fibroblast populations that have previously been associated with prognosis^26^. Immune infiltrate status is on the X axis for HR (red) and TN (blue)with the GSVA score on the Y axis. Panels show the vascular (vCAF, A), extracellular matrix (mCaf, B), developmental (dCAF, C), and cell cycle related (cCAF, D) fibroblast populations.

Many different populations of cancer-associated fibroblasts have been identified in breast cancer. Four populations that have been associated with prognosis in breast cancer in the TCGA include cell cycle related (cCAF), developmental (dCAF), extracellular matrix (mCAF), and vascular (vCAF) cancer associated fibroblasts. The vCAF signature was an independent prognostic indicator of developing metastatic disease. In the immune-high populations, the HR+ HER2− tumors expressed higher vCAF fibroblast signature than the immune high TN tumors (*P*-value = 1.7×10^− 11^). In the immune-low, the same trend was seen but there a less significant difference (*P*-value = 7.8×10^− 3^) (Fig. 5A). The immune-high HR+ HER2− tumors also had higher mCAF expression than the TN tumors (*P*-value = 2.1×10^− 13^). This mCAF signature has also been associated with increased risk of disseminated disease (Fig. 5B).[28] TN tumors had higher cCAF and dCAF expression than HR+ HER2− tumors in both immune-high and immune-low populations (Fig. 5C and D). These fibroblast types are not associated with tumor outcome. [28] There was an association between higher vCAF and lower TH1 tumor immune infiltrate in both the HR + HER2− immune-high (ρ=−0.6 *P*-value < 2.2×10^− 16^) and HR + HER2− immune-low (ρ=−0.41 *P*-value = 6.5×10^− 13^) and the TN immune-high (ρ=−0.36 *P*-value = 0.00065) but not in TN immune-low (ρ=−0.18 P-value = 0.3) (**Fig. S6**_**A**_**, S6**_**B**_). There was also a significant negative association of fibroblasts to TH1 immune infiltrate in both the HR + HER2− immune-high (ρ=−0.63 *P*-value < 2.2×10^− 16^) and HR + HER2− immune-low (ρ=−0.45 *P*-value = 1.6×10^− 15^) as well as TN immune-high (ρ=−0.42 *P*-value = 6.9×10^− 5^) and TN immune-low (ρ=−0.44 *P*-value = 0.0075) (**Fig. S6**_**C**_ **and S6**_**D**_).

## Discussion

Gene expression analysis has identified subtypes of human breast cancer and can be used to guide treatment[37] but the differences in immune environment between the subtypes and how to address them in breast cancer remains unknown with relatively few breast cancer patients benefiting from immune based therapies. Evaluating immune gene clustering in TN and HR + HER2− breast cancer, the breast cancers did not stratify into canonical subtypes but rather divided into immune-high and immune-low tumors containing both subtypes. These immune-high and immune-low groups could be seen in two independent datasets, the TCGA and METABRIC.[38, 39] Evaluation of the immune-high TN and HR + HER2− tumors demonstrated that they did not have significant differences in several common signatures associated with response to ICI and that the HR + HER2− immune high tumors contained both the best and worst prognostic immune environments. The largest differences in expression between immune high TN and HR + HER2− tumors were that fibroblasts, specifically mCAF and vCAF that are associated with worse prognosis in breast cancer and were highest in HR + HER2− tumors while expression of TH1 and TH2 genes were highest in TN tumors.

We compared several signatures that are associated with increased immune response and improved response to ICIs including CD8 + and T_reg_ signatures and the IFNγ signature.[40] The IFNγ^+^ gene signature can identify tumors that respond to anti-PD-LI ICI durvalumab in 304 NSCLC patients and 103 urothelial cell cancers independent of PD-L1 expression.[6] The CD8 + and Treg signatures have been associated with improved response to immune checkpoint inhibitors in non-small cell lung cancer and urothelial cancer.[6, 7] However, while all of these signatures were higher in the immune-high breast tumors, there were no significant differences between HR + HER2- and TN immune-high tumors. In immune-low tumors both CD8+, and T_reg_ signatures were higher in TN compared to HR + HER2− (Fig. 3D). There was no difference between CD8 + and T_reg_ signatures between immune-high HR + HER2- and TN tumors and we found HR+ HER2- and TN immune-high tumors to have similar ranges of IFNγ^+^ signature GSVA scores (Fig. 4A). In immunogenic tumors such as melanoma or NSCLC, neo-antigens were associated with T cell mediated anti-tumor responses. Increased mutational load predicted a higher rate of response to anti-CTLA4 in melanoma and anti-PD-1 in lung cancer.[41, 42] There was higher TMB in TN verses HR + HER2− tumors (P-value = 3.16×10^− 15^). Furthermore, comparing the immune high TN and HR + HER2− tumors, there was more TMB in the TN immune high tumors (P-value = 3×10^− 5^). This might explain the increased TH1 and TH2 immune infiltrate in the TN immune high tumors as compared to the HR+ HER2− immune high tumors (Fig. 4B) explaining the increased adaptive immune reactivity in TN immune-high tumors.

Six common immune environments can be seen across 33 diverse tumor types and determine tumor prognosis and response to therapy[27], with the C3 immune environment having the best clinical outcome. In this study, there was only six immune high TN tumors with a C3 immune environment therefore the majority of immune-high C3 tumors were HR + HER2−. Most immune-high TN tumors were the C2 immune subtype (Fig. 2B_i_). The C6 tumors predict the worst prognosis with increased TGF-β expression and are associated with increased tumor cell migration and activation of immune suppression from the tumor stroma in breast cancer.[43, 44] Only HR+ HER2− immune-high tumors had the C6 immune environment (Fig. 2B_ii_). In the immune-high group, HR + HER2− breast cancer contained the best prognosis (C3) and worse prognosis (C6) tumors supporting that there are differences between immune-high TN and HER2 + HER2− tumors that may explain why they have differential prognosis and response to ICI. We observed two major differences between HR + HER2- and TN within the immune-high tumors, TN immune-high tumors were enriched for TH1 and TH2 T cells while HR + HER2− immune-high tumors were enriched for fibroblasts, and TN immune-high tumors had increased TMB as compared to immune-high HR+ HER2− tumors (Fig. 3C, 4B).

The increased expression of CAF in HR + HER2− immune-high breast tumors may suggest a mechanism that prevents these tumors from developing an appropriate anti-tumor immune response. The interaction between the tumor and stroma is important in determining the breast cancer subtype that develops.[45, 46] The tumor stroma further can determine metastatic potential[47], resistance to chemotherapy[48], and modulating the immune response to the tumor[46]. When looking at breast cancer as a whole, expression of stromal and fibroblast components have been associated with worse prognosis including PDGFRA, PDGFRB, CXCL1, CXCL14, CD10, and CD36.[49] CAFs have been associated with mesenchymal stem cells and inducing a wound healing response associated with immunosuppression.[50] In breast cancer, CAF expression has been correlated with decreased CD8 + T cells and increased macrophages. [51] Of interest, in a recent study of human breast cancer samples, four CAF subtypes were identified and the vascular (vCAF) and mesenchymal (mCAF) subtypes associated with developing metastatic disease.[28] In the TCGA dataset, both of those subtypes of CAF are significantly increased in the HR + HER2− breast tumors as compared to the TN breast tumors and there is higher significance in the immune-high population (Fig. 5A and B). In this study we were unable to evaluate whether CAFs induce immunosuppression in immune-high HR + HER2− tumors but further evaluation of CAF in patients that do not respond to ICI is needed.

There are a few limitations in this study. The dataset lacks adequate tumor stage information thus we could not investigate the relationship between our findings and the stage of breast cancer.

Previous studies have shown that more advanced tumors have decreased immune infiltrate and increased immunosuppression.[52] Most importantly, with the lack of equivalent -omics and outcome data on ICI treated tumors, we could not evaluate the efficacy of ICIs on immune-high and -low tumors or compare ICI outcomes between HR + HER2- and TN tumors. While there have been some data showing benefit in HR+ HER2− patients receiving neoadjuvant pembrolizumab with chemotherapy, the magnitude of response was 24.3% pCR compared to 64.8% pCR with addition of pembrolizumab to chemotherapy in TN patients. [19, 20] Still, the biomarkers that predict response to ICI evaluated showed no significant differences between HR+ HER2- and TN in the immune-high group: HR + HER2- and TN have similar expression of CD8 + and T_reg_ expression while immune high TN does have higher TMB. We do find immune-high HR+ HER2− tumors have increased CAF while immune-high TN tumors have increased helper T cells and increased expression of T cell modification genes. Further studies will need to be performed to determine whether these differences may affect how HR + HER2- and TN immune-high tumors respond to ICI.

## Figures and Tables

**Table 1:** RNA sequencing cell types abundance estimation

Class	Cell type Note
Dendritic	aDC
	cDC
	DC
	iDC
	pDC
B cells	B cells
	Class-switched memory B cells
	Plasma cells
	proB cells
	Neutophils
NK cells	NK
	NKT
CD4 + T cells	CD4 + T cells
	CD4+ naïve T cells
	CD4 + memory T cells
	CD4 + effector memory T cells
	CD4 + central memory T cells
CD8 + T cells	CD8 + T cells
	CD8 + naïve T cells
	CD8 + effector memory T cells
	CD8 + central memory T cells
	Th1 cells
	Th2 cells
	Treg cells
	Tgd cells
	M1 macrophages
	M2 macrophages
	Monocytes

## Data Availability

All data generated or analyzed during this study are included in this published article and its supplementary information files.

## Abbreviations

TIL: Tumor infiltrating lymphocytes
HR+, HER2−: Hormone receptor positive
HER2+: HER2 positive
TN: Triple negative
ICI: Immune checkpoint inhibitor
FC: Fold change
GSVA: Gene set variation analysis
CAF: Cancer associated fibroblast
